# Assessment of population structure and genetic diversity of German Angora rabbit through pedigree analysis

**DOI:** 10.5713/ab.22.0228

**Published:** 2022-11-14

**Authors:** Abdul Rahim, K. S. Rajaravindra, Om Hari Chaturvedi, S. R. Sharma

**Affiliations:** 1 North Temperate Regional Station, ICAR-Central Sheep and Wool Research Institute, Garsa, Kullu (H.P.) 135141, India; 2 ICAR-Directorate of Poultry Research (DPR), Rajendranagar, Hyderabad 500 030, Telangana, India; 3 ICAR-Central Sheep and Wool Research Institute, Avikanagar, Rajasthan 304501, India

**Keywords:** Angora Rabbits, Diversity, Effective Population Size, Generation Interval, Genetic Structure, Inbreeding

## Abstract

**Objective:**

The main goals of this investigation were to i) assess the population structure and genetic diversity and ii) determine the efficiency of the ongoing breeding program in a closed flock of Angora rabbits through pedigree analysis.

**Methods:**

The pedigree records of 6,145 animals, born between 1996 to 2020 at NTRS, ICAR-CSWRI, Garsa were analyzed using ENDOG version 4.8 software package. The genealogical information, genetic conservation index and parameters based on gene origin probabilities were estimated.

**Results:**

Analysis revealed that, 99.09% of the kits had both parents recorded in the whole dataset. The completeness levels for the whole pedigree were 99.12%, 97.12%, 90.66%, 82.49%, and 74.11% for the 1st, 2nd, 3rd, 4th, and 5th generations, respectively, reflecting well-maintained pedigree records. The maximum inbreeding, average inbreeding and relatedness were 36.96%, 8.07%, and 15.82%, respectively. The mean maximum, mean equivalent and mean completed generations were 10.28, 7.91, and 5.51 with 0.85%, 1.19%, and 1.85% increase in inbreeding, respectively. The effective population size estimated from maximum, equivalent and complete generations were 58.50, 27.05, and 42.08, respectively. Only 1.51% of total mating was highly inbred. The effective population size computed via the individual increase in inbreeding was 42.83. The effective numbers of founders (f_e_), ancestors (f_a_), founder genomes (f_g_) and non-founder genomes (f_ng_) were 18, 16, 6.22, and 9.50, respectively. The f_e_/f_a_ ratio was 1.12, indicating occasional bottlenecks had occurred in the population. The six most influential ancestors explained 50% of genes contributed to the gene pool. The average generation interval was 1.51 years and was longer for the sire-offspring pathway. The population lost 8% genetic diversity over time, however, considerable genetic variability still existed in the closed Angora population.

**Conclusion:**

This study provides important and practical insights to manage and maintain the genetic variability within the individual flock and the entire population.

## INTRODUCTION

Rabbit farming is a profitable business with tremendous scope for improving the living standard of small and marginal rural farmers [1]. According to 20th livestock census of the Government of India, total rabbit population declined from 0.592 million in 2012 to 0.550 million in 2019. German Angora is primarily reared for fine wool production as its wool production potential is much higher and priced 10 to 30 times more than that of sheep [2]. The wool obtained from Angora rabbits is preferred over other wools due to its fineness (12 to 16 μm), softness, silky texture, fluffiness, lack of odor and anti-static property to repel the dirt [3]. It is much warmer (eight times) and lighter than sheep wool due to the hollow core of the Angora fiber and is used either in pure or blended form for making garments [4].

Knowledge of genetic variability is very essential to evaluate the population under selection in every generation and is a prerequisite for deciding and formulating effective breeding strategies [5]. Genetic variation is considered the primary biological resource that can be exploited in future breeding programs [6]. Some genetic parameters are highly influenced by management and mating systems, resulting in a severe loss of genetic diversity (GD). Inbreeding in farm animals is generally challenging to avoid due to farming practices. The risk of inbreeding also increases with higher selection intensity and smaller population sizes. Heterozygosity and allelic variations could be rapidly lost in small, closed, and selected populations. Pedigree analysis is one of the most economical, easiest, and most efficient tools to assess demographic parameters and genetic variability of the population, in contrast to employing molecular data [7]. Populations under long-term selection programs tend to change in their initial structure over time and such change can be analyzed through pedigree information. Incomplete pedigree information after generations leads to overestimation of effective population size.

Gene origin statistics provide a historical perspective of changes occurring in a population. The probability of gene origin is used to identify the extent to which individuals influence the genetic history of a population. Inbreeding and effective population size is useful for long-term management of genetic variability and monitoring of genetic trends. Also, the knowledge of population structure and its genetic changes could assist in future management decisions, allowing for policies for genetic improvement and adaptation of a breed to a specific region [8].

German Angora flock was established in 1986 at North Temperate Regional Station (NTRS), ICAR-Central Sheep and Wool Research Institute (CSWRI), Garsa, India. In 1997, superior germplasm from Germany was introduced and maintained as a closed flock. The population was well acclimatized to the Himalayan terrain of Himachal Pradesh. The flock was genetically improved over the last 24 years covering 22 generations of selection. The germplasm was subjected to genetic selection for improvement of production performance. Nationally, this station has been recognized as a germplasm centre for pedigreed German Angora rabbits. The superior germplasm was made available to state Animal Husbandry department, farmers, different developmental agencies, and private entrepreneurs. Genetic variation and relatedness among the breeds are prerequisite information because genetic variability is considered the primary biological resource that can be utilized in future breeding programme. There are no studies available on population structure and GD in German Angora rabbits. Therefore, the present investigation was carried out to assess the population structure and GD of a nucleus flock of the German Angora rabbits through pedigree analysis.

## MATERIALS AND METHODS

### Data collection and management practices

The pedigree information of 6,145 German Angora rabbits born between 1996 to 2020 were collected from the Angora rabbit unit maintained at NTRS ICAR-CSWRI, Garsa, Kullu (Himachal Pradesh) India. The farm is located at 31.28°N latitude and 77.20°E longitude with an altitude of 1,400 to 2,100 meters above average sea level in the north temperate Himalayan valley of Himachal Pradesh. The climate is sub-temperate where the temperature ranges from −4°C to 35°C with an average annual rainfall of about 840 mm, mainly during the monsoon season. The superior germplasm was introduced in 1997 by importing 40 bucks and 60 does of German Angora rabbit from Germany. The flock was subsequently closed to outside breeding, where approximately 40 to 60 breeding does were maintained yearly with a male to female ratio of 1:5. The management system was fully intensive with the provision of clean drinking water and *ad libitum* feeding in the morning and evening. Animals were fed seasonal grasses *ad libitum* and concentrate (15% to 20% crude protein) in graded quantities ranging from 90 to 220 g according to their age and physiological status. The lactating doe and kits were kept together in the kindling cage until weaning at 42 days of age. The weaned kits were transferred to individual wire mesh cage under similar housing and management practices. Each cage was equipped with the steel or earthen bowls for offering the concentrate feed and water. Sexing and ear tagging were done at the time of weaning. The rabbits were mated as and when they attain sexual maturity at 6 to 7 months of age. The female rabbit was brought to the cage of her assigned buck for breeding and returned to her cage after mating. In case of a failed conception, the doe was remated to the same breeding buck after pregnancy diagnosis. Mating of closely related individuals was avoided to keep the inbreeding levels to as minimum as possible. Symptomatic treatment was adopted for disease management under the direction of a veterinarian.

### Pedigree analysis

Data from 6,145 German Angora rabbits born between 1996 and 2020 were used for pedigree analysis and population structure characterization. The pedigree of these animals was traced as far back as possible in the pedigree register maintained at the farm. The analysis included all the ancestors and relatives of each individual. Animals with both known parents were used as the reference population for estimation of gene origin statistics using ENDOG v4.8 program [9].

### Pedigree completeness

The pedigree completeness index (PCI) was calculated to provide information about the quality of the pedigree. The PCI was computed based on the completeness of the pedigree in the previous generations as described by MacCluer et al [10].


$${\text{I}}_{d}=\frac{4C_{sire}C_{dam}}{C_{sire}+C_{dam}}$$

where *C_sire_* and *C_dam_* indicate the contribution from the sire and dam lines, respectively. The contributions were computed from the following formula [10].


$$C=\frac{1}{d}\sum_{i=1}^{d}a_{i}$$

where *a_i_* indicates the percentage of ancestors known in the ith generation and d is the total number of generations taken into consideration for the calculation of pedigree completeness. In this study, when calculating the average PCI for each individual according to the birth year, complete pedigrees were considered up to fifth generations of ancestors. EVA software was used to compute this index [11].

For each individual, following parameters were estimated to avoid introduction of individuals with incomplete pedigree records. The maximum number of generations indicates the number of generations between the individual and its most distant ancestor. The equivalent generation indicates the number of generations between the individual’s second-generation ancestors and the offspring of the furthest generation. Ancestors with unknown parents were considering as founders (generation 0). The complete generation was estimated by averaging over the sum of (1/2)^n^ of all known ancestors [12]. Where n represents the number of generations separating the individual from each known ancestor (parent = 1, grandparent = 2, and so on).

### Generation interval

The generation interval (GI) is the mean age of the parents at the time of birth of their progeny. The GI was computed by considering the four-selection pathway model, sire to buck (L_sb_), sire to doe (L_sd_), dam to buck (L_db_), and dam to doe (L_dd_). The mean GI of the population was calculated as the average of the four pathways according to Falconer and Mackay [13].


$$GI=\frac{L_{sb}+L_{sd}+L_{db}+L_{dd}}{4}$$

### Inbreeding coefficient (F) and average relatedness

The inbreeding coefficient is the probability that two alleles at a locus in an individual are identical by descent [14]. The F was determined using the algorithm proposed by Meuwissen and Luo [15]. The individual increase in inbreeding (Δ*F_i_*) was computed using the basic formula described and modified by Gonzalez-Recio et al [16–18].


$$\Delta F_{i}=1-\sqrt[{t_{i}-1}]{1-F_{i}}$$

where *F_i_* is the coefficient of inbreeding for the individual *i* and *t_i_* is the corresponding equivalent complete generations. For each individual, the number of equivalent complete (EqG) generations was determined as


$$E_{q}G_{i}=\sum {\left(\frac{1}{2}\right)}^{n}$$

where n is the number of generations between each known ancestor and the sum is computed across all known ancestors of ith individual [12].

The average relatedness (AR) is used as a complement or alternative to the coefficient of inbreeding to predict the long-term inbreeding of a population. The AR is the probability that an allele selected at random from the whole population belongs to a given animal [19]. Hence, AR was computed as an average of the coefficients in the row corresponding to the individual in the numerator relationship matrix [20]. It can thus be interpreted as the demonstration of the animal in the whole pedigree despite the information of its own pedigree.

### Effective population size (N**_e_**)

The effective population size (N_e_) is the number of breeding individuals that would give rise to calculated sampling variance or rate of inbreeding (ΔF) in the form of an idealized population. N_e_ was estimated using the individual increase in inbreeding coefficients [17,18].


$$\Delta F_{i}=1-\sqrt[{EqG_{i}-1}]{1-F_{i}}$$

where *E_q_G_i_* and *F_i_* are equivalent complete generation and coefficient of inbreeding for an individual *i*. The individual increase in inbreeding coefficients was averaged and N_e_ was calculated as described by Falconer and Mackay [13].


$$N_{e}=\frac{1}{2\Delta F}$$

Accordingly, N_e_ was computed by individual inbreeding coefficients regressed on the equivalent generations, complete and maximal generations traced as described by Maignel et al [12].

### Gene origin probabilities

The genetic background in terms of the probability of gene origin was determined by computing the various parameters as explained by Boichard [21]. The effective number of founders (*f_e_*) was calculated as the number of founders that would be expected to contribute equally with genetic material to produce the same GD as the population under consideration [22]. The *f_e_* was calculated from the following formula.


$$f_{e}=\frac{1}{\sum_{k=1}^{f}q_{k}^{2}}$$

where, *f* is the total number of founders and *q_k_* is the estimated proportional genetic contribution of founder k as determined by the founder’s average relationship to each animal in the current population.

The effective number of ancestors (*f_a_*) is the minimum number of individuals (founders or non-founders) required to explain the complete GD of the current population. *f_a_* was computed to assess the population bottlenecks [21].


$$fa=\frac{1}{\sum_{j=1}^{f}q_{j}^{2}}$$

where *f_a_* indicates the total number of ancestors and *q_j_* indicates the marginal contribution of jth ancestor.

The marginal contribution is the genetic contribution produced by an ancestor that could not be explained by another ancestor previously selected [23]. The founder genome equivalent (f_g_) accounts for genetic variation that may be lost due to random drift in small populations despite an equal contribution of all the founders in the population [22]. The inverse of twice the average co-ancestry between individuals in the reference population was used in the calculation of *f_g_*.


$$f_{g}=\frac{1}{2}\bar{f}$$

where, *f* indicates the average co-ancestry between participants in the reference population. The *f_g_* should be smaller than both *f_a_* and *f_e_*, which would account for all factors that influence gene loss during segregation. The non-founder genome equivalent (*f_ng_*) accounts for GD loss due to genetic drift accumulated over non-founder generations. The *f_ng_* was computed according to Caballero and Toro [24].


$$\frac{1}{f_{ng}}=\frac{1}{f_{g}}+\frac{1}{f_{e}}$$

The genetic bottleneck was determined by calculating the number of ancestors in the population that contributed to 50 percent of the genes (*f_a50_*) and the ratio of *f_e_*/*f_a_*. The *f_a_* is expected to be smaller than the *f_e_* in the presence of a bottleneck, which can be indicated by *f_e_*/*f_a_* ratio.

### Genetic diversity

The degree of genetic variation in the reference population in comparison to that existing in the base population was estimated by calculating the Nei expected heterozygosity. Genetic diversity was computed according to Lacy [22,25].


$$GD=1-\frac{1}{2f_{g}}$$

Genetic diversity in the base population was calculated as:


$${GD}^{*}=1-\frac{1}{2f_{e}}$$

The difference between GD* and GD was computed as per Caballero and Toro [24].


$${GD}^{*}-GD=\frac{1}{2f_{ng}}$$

The genetic diversity lost in the founder generation was estimated by 1-GD. The genetic diversity loss by unequal distribution of founder’s alleles was estimated by 1-GD* using the method of Caballero and Toro [24].

### Genetic conservation index

Genetic conservation index (GCI) was estimated from the genetic contributions of all the identified founders of the reference population as described by Alderson [26].


$${GCI}_{i}=\frac{1}{\sum {pj}^{2}}$$

where *p_j_* is the percentage of genes of jth founder contributed to the pedigree of ith animal.

The analysis of data generated on German Angora rabbits was carried out using the software package ENDOG 4.8 to estimate the genetic diversity and population structure in a well-organized farm [19].

## RESULTS AND DISCUSSION

### Pedigree statistics

**Figure 1:**
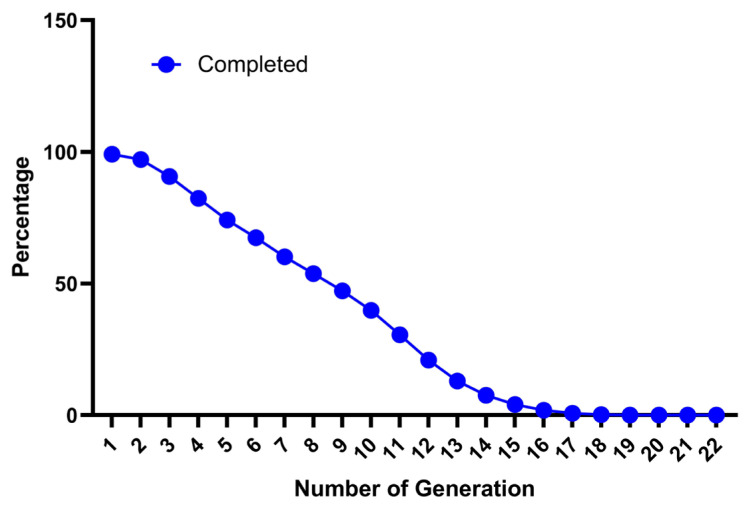
Percentage of known ancestors per generation (pedigree completeness) for the whole population of the German Angora rabbit.

German Angora is a fine fibre Angora rabbit breed maintained in a close flock at NTRS, ICAR-CSWRI, Garsa, Kullu (Himachal Pradesh). Due to the closed nature of the flock and the inability to introduce fresh germplasm from outside it is expected that the flock may have high incidence of inbreeding. The present study revealed that the pedigree data was well maintained and provided meaningful information about the genetic architecture of the German Angora rabbit. The statistical analysis of pedigree data of the German Angora rabbit is presented in Table 1. Analysis revealed that, 99.09 percent of rabbits had known pedigree information indicating a high degree of pedigree completeness. The completeness level for the whole pedigree was reduced with the latest generations as 99.12%, 97.12%, 90.66%, and 82.49% for the 1st, 2nd, 3rd, and 4th generations, respectively (Figure 1). A study on broiler rabbits by Sakthivel et al [27] also reported a similar pattern of reduction of completeness from 98% to 71% within the first four generations. Another study also suggests a higher estimate up to fifth generation (94.50%) and a lower (82.10%) estimate up to the tenth generation in synthetic rabbits [28]. Another study also suggested a decrease from 100 to 94.50 percent up to the fifth generation and 82.10 percent up to the tenth generation of the whole pedigree population for the synthetic Pannon White rabbits [28]. The pedigree of the present generations was more detailed as compared to the older generations. The percentage of ancestor knowledge was balanced, with almost equal proportions for sire and dam pathways when considering recent generations. This might be due to the importation of rabbits with an almost equal sex ratio (2:3 buck to doe ratio), abilities to have multiple births, induced ovulation and maintenance of a closed population. A higher percentage of PCI indicates that the pedigree records of German Angora are well maintained in the institute’s regional database. The maximum number of known generations was 22 in the present investigation. The generation-wise decrease in percentage of ancestral information in whole pedigree population is shown in Figure 1. The first ancestral generation was 99.12% complete and then it decreased progressively for subsequent generations. More than 10% of ancestors were traced up to 13 generations. However, very few individuals had known ancestors from the fourteenth generation onward, indicating that getting pedigree information beyond that was difficult. Similarly, a decreased percentage of known ancestors in subsequent generations were also reported in New Zealand white rabbit and different exotic and native sheep breeds of India [5,27,29]. In a breeding population, pedigree completeness up to known generations is important to get reliable estimates of inbreeding, gene flow and other factors.

**Figure 2:**
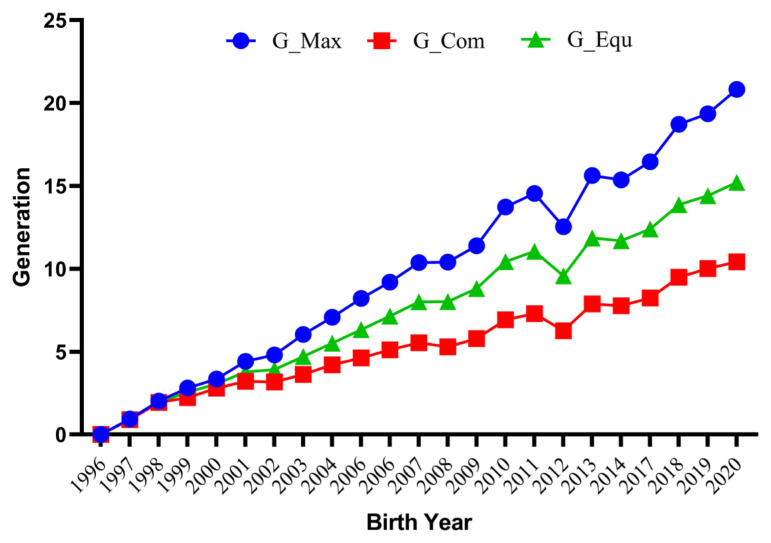
Annual trends for maximum (G_Max), equivalent (G_Equ), and complete (G_Com) generations traced for the whole pedigree in German Angora rabbits.

The average number of maximum generations, equivalent generation and complete generations for the studied population were 10.28±0.07, 7.91±0.05, and 5.51±0.03, respectively (Table 2). The maximum values for these estimates were 22.00, 16.07, and 11.00, respectively. These values were comparatively higher than those reported by Sakthivel et al [27]. However, the average equivalent generation was lower than the value reported (11.36) for Pannon White rabbits [28]. The mean maximum generations, equivalent generations and complete generations for the whole pedigree traced by year of birth are depicted in Figure 2.

### Inbreeding and average relatedness

**Figure 3:**
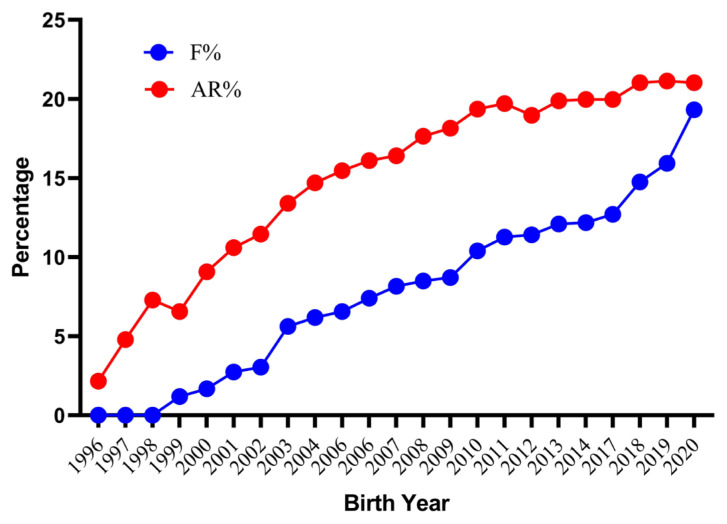
Trends for inbreeding coefficient (F) and average relatedness (AR) of German Angora rabbits by year of birth for the whole pedigree.

The average inbreeding coefficients and AR for the whole analyzed pedigree were 8.07% and 15.82% respectively. The inbred animals had an average inbreeding coefficient of 9.30%. However, females (9.33%) had a slightly higher inbreeding coefficient than males (9.27%). The lower estimate of the inbreeding coefficient has also been reported in three different populations of Pannon (5.54%, 6.30%, and 7.69%), Botucatu (7%) and Sika (6.5%) rabbits [28,30,31], whereas higher values has been reported in New Zealand white (13.23%) and Ibicenco (10.80%) rabbit [27,32]. With the closed nature of the flock and good pedigree depth resulted in an observed AR 7.75 percent higher than the inbreeding coefficient. A higher AR combined with a lower inbreeding coefficient indicates a high degree of relatedness among all individuals of pedigree. This could lead to difficulties while trying to avoid mating between unrelated or distantly related individuals. About 1.51% of total mating was highly inbred, out of which 0.10% was full-sib matings, 0.78% was half-sib matings and 0.63% was parent-offspring matings. The frequency of highly inbred mating in present population was low in comparison to an earlier report on New Zealand white rabbits [27]. The trends for the inbreeding coefficient and AR by year of birth are shown in Figure 3. The mean value of the inbreeding coefficient increased considerably over the years reaching the peak value of 19.32% for current population. The present findings are similar to those in Pannon and New Zealand white rabbits, in which an increasing trend over the years has been reported [27,28]. Further investigation into records from 2018 to 2021 reveal that the inbreeding values were higher due to mating of a lesser number of bucks contributing to a greater number of kits due to the breeding ban instituted by the Government of India between 2014 and 2017. In August 2017, the breeding was resumed from few older rabbits to increase the flock strength. The number of rabbits was relatively stable up to 2013 with average flock strength of 500 rabbits. From 2014, this flock strength considerably decreased and reached less than 50 rabbits in 2017 and very few animals were able to reproduce in the next generation. The average inbreeding coefficient, AR, and percentage of inbred individuals with their inbreeding per complete generation are shown in Table 3. The percent inbreeding increased gradually from 1.12% in the second generation to a maximum of 21.70% in the eleventh generation. The proportion of inbred animals in the second generation was only 30.15 percent, which rapidly increased to become 100% in the fifth generation, where the inbreeding coefficient and mean inbreeding of the inbred population became the same. The present results are comparable to those reported earlier in New Zealand white rabbits [27]. However, the rate of inbreeding is more important than the absolute values of the inbreeding coefficient [33]. The estimated rate of inbreeding was less than the critical values (<1% per generation) except for animals born in 1998, 2000, 2002, 2009, and 2017 to 2019. As per the recommendation by Bijma and Wooliams [34], a rate of inbreeding of more than one percent per generation should be avoided to maintain fitness traits in a breed. The percent increase in inbreeding over generations was 0.85, 1.19, and 1.85 predicted through maximum generations, equivalent generations, and complete generations, respectively (Table 4). Inbreeding in the current study was distributed from low to high range in a wide array of individuals and was comparable with the findings of Nagy et al [28] in Pennon white rabbits. Our additional investigations indicate a maximum inbreeding of 36.96% in an individual in the current subpopulation.

### Effective population size (N**_e_**)

**Figure 4:**
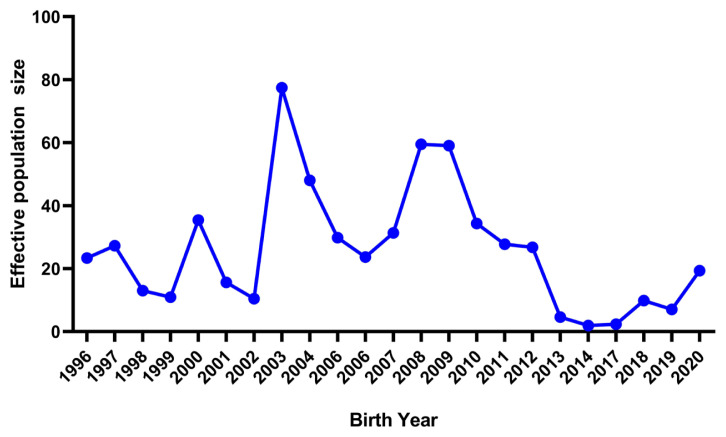
Annual trend for effective population size estimated by variances of family sizes in German Angora rabbits.

The realized N_e_ computed via the individual increase in inbreeding was 42.83 in the present population. The N_e_ estimates measured from the pedigree information are presented in Table 5. N_e_ computed from regression and log regression analyses on equivalent generations was 38.85 and 38.43 and those on the birth date were 39.29 and 38.49, respectively. The N_e_ estimated from equivalent generations, maximum generations and complete generations was 27.05, 58.50, and 42.08, respectively. However, these N_e_ values are quite higher than the estimates reported by Sakthivel et al [27] in a closed population of broiler (New Zealand white) rabbits. In a similar study, Ne ranged from 29 to 47 in low and high lines of Angora rabbits [35]. Similarly, Nagy et al [28] reported N_e_ from 37.19 to 91.08 in a closed population of Pannon white rabbits. The trends of N_e_ over the years estimated by the variances of family sizes are depicted in Figure 4. The N_e_ estimates showed a fluctuating trend over the year’s up to 2012 and dropped suddenly in 2014 due to the stoppage of breeding and again showed an increasing trend from 2017 onwards after the resumption of breeding from the same flock. However, the values of N_e_ were not constant and changed with the passage of time according to the level of inbreeding. This situation can get amplified with the increasing trends of inbreeding. N_e_ in the present study is far below the critical value recommended by Food and Agriculture Organization [36]. Normally, the value should lie between 50 to 100 and should not fall below 50 to sustain the genetic diversity for conservation and selection programs [33,36]. The lower estimates of N_e_ indicate that the present flock is at risk for the instant effects of inbreeding depression. Thus, the population is under threat from reduction of adaptive genetic variation and difficult to improve through selection.

### Generation interval

In the present study, the GI estimates for the four-selection pathways were 1.75 (Sire-buck), 1.65 (Sire-doe), 1.37 (Dam-buck) and 1.32 years (Dam-doe), giving an average value of 1.51 years (Table 5), which is comparable with earlier reports of Rafat et al [35] who also reported an average GI of 1.54 years in low line and 1.64 years in the high line of Angora rabbits in France. Similarly, Sakthivel et al [27] reported GI values of 1.36 (Sire-buck), 1.53 (Sire-doe), 1.44 (Dam-buck), 1.59 years (Dam-doe) with an average of 1.49 years across the four pathways in New Zealand white rabbit. Nagy et al [28] reported a lower GI value as 1.12 years in Pannon White rabbits than the present study. Estimation of GI is essential to breeding programs as it directly affects the response to selection for the traits under selection. The estimates of GI vary from flock to flock in a breed, which might be due to variation in different agro-climatic conditions, genetic structure of population and management practices. Lower GI enhances the annual genetic gain for the targeted traits, as it measures the time required for genes to be passed from parents to their offspring. However, according to Santana and Bignardi [37] long GI minimize the annual inbreeding rates, consequently increasing the N_e_ and preserving the genetic structure of the population. In our study, GI for sire-offspring pathways was longer relative to the corresponding value for dam-offspring pathways, in agreement with the findings of Nagy et al [28] in Pannon white rabbits. This could be due to the continuous use of few superior sires for a prolonged period.

### Probability of gene origin

The parameters describing the probability of gene origin in the German Angora rabbit were calculated using animals with both parents known, which we defined as the reference population (Table 6). The total number of animals, number of ancestors and founders contributing to the reference population were 5,946, 61, and 56, respectively. The effective number of founders (*f_e_*) and ancestors (*f_a_*) was 18 and 16, respectively, indicating that the reference population had somewhat similar genetic diversity as the founders. A lower estimate than the current study was reported in New Zealand White rabbits as 10 and 11, respectively [27]. However, a higher estimate of 48 and 26 was reported by Nagy et al [28] for Pannon White rabbits. The values of *f_e_* and *f_a_* are generally higher in larger populations, particularly when the size of the founder populations in the beginning was also high [38]. The *f_e_*/*f_a_* ratio was 1.12, indicating that founder contributions were unequal and only modest bottlenecks occurred in the population. A comparable ratio of 1.10 was also reported in New Zealand white rabbit [27]. Ideally, *f_e_*/*f_a_* ratio would be one and any deviations indicate unbalanced use of sires posing a significant risk for the loss of original genetic diversity. However, in the present study, *f_e_*/*f_a_* was greater than one indicating occasional genetic bottlenecks occurred in the flock. Bottlenecks are likely to have occurred, when *f_a_* was larger than *f_e_*, and the *f_e_*/*f_a_* ratio, resulting in a more intense bottleneck [39]. The *f_e_*/*f_a_* ratio is used to determine how much genetic diversity lost in the founders is a result of bottlenecks [23].

Numbers of ancestors explaining 100, 75, and 50 percent genetic diversity of the gene pool were 61, 13, and 6 respectively. The six most influential ancestors explained half the genetic diversity of the population, whereas the most influential ancestor solely contributed to 7.75% of the variations. Similarly, four ancestors accounted for 50% of the genetic variability in a New Zealand white rabbit population [27]. Different estimates obtained in different populations may be explained to a great extent by different genealogical structures of the studied flocks in terms of pedigree completeness, different mating and management policies and excessive use of preferential elite males for breeding. The estimated founder genome equivalents (*f_g_*) and non-founder genome equivalents (*f_ng_*) values in the present population were 6.22 and 9.50, respectively. The *f_g_* is linked to genetic variability loss caused by genetic drift in subsequent generations, whereas the *f_ng_* calculates the amount of genetic drift that has occurred across the population since its inception. The lower *f_g_* estimate indicates a small portion of founder’s genes are present in the population. The loss of genetic variability was also reported to be higher when *f_e_* estimates became higher than *f_ng_* due to accumulation of the genetic drift in the non-founder generation [40].

### Genetic diversity

The estimated value for GD in the reference population relative to the base population or Nei expected heterozygosity was 0.920, indicating that approximately 8% of the genetic diversity in the base population was lost during the study period. However, the genetic diversity estimated for the base population (GD*) was 0.985. In the founder generation, losses in heterozygosity due to genetic drift and bottleneck effects (1-GD) and uneven contributions of founder alleles (1-GD*) were calculated as 0.080 and 0.015, respectively. The present findings are in accordance with the earlier reports in Adani goats [40] and Muzaffarnagri sheep [41], which reported loss of 3% and 3.2% genetic diversity, respectively, in the base population. In most cases, *f_g_* is observed to be less than the values of *N_e_*, *f_e_*, and *f_a_* and it compensates for unequal founder contributions as well as fraction of founder genomes lost from the pedigree by genetic drift during bottlenecks. Unequal contributions of the founder gene are confirmed by the low estimates of *f_g_* in the present reference population. Further, the difference between *f_g_* and *f_a_* also revealed the random loss of alleles from founder animals and small percentage of founder genes in the reference population. According to the *f_g_*/*f_a_* ratio, the reference population still retained 39.05% of its ancestral genetic diversity. In the present Angora population, *f_e_* was observed to be lower than *f_ng_*, implying that the loss in genetic variability was mostly due to genetic drift accumulated in non-founder generations.

### Genetic conservation index

The average GCI and their corresponding frequency distribution are presented in Tables 6 and 7. The average estimate of GCI was 13.91% in all animals with the males (14.02%) recording a higher GCI than the females (13.79%). The average GCI in the present investigation was higher than the reports in New Zealand white rabbits [27]. Mean values of GCI improved continuously over time and reached a maximum value of 18.57, which was estimated in three individuals. The increase of GCI over time indicates that there was no introduction of germplasm from outside in the nucleus flock. The main objective of GCI is based on the need to conserve the entire spectrum of alleles that exist in the base population for conservation purposes. Individuals would normally receive equal contributions from all the original ancestors in the population, resulting in higher animal conservation values. Higher the GCI value, the more valuable an animal is in terms of conservation. In the current population, the number of animals with GCI greater than 15 was 60.75% while more than 18 animals had a maximum CGI value of 0.98% (Table 7). This could be used as a tool to guide selection in a breeding flock and mating in conservation programs.

## CONCLUSION

The present German Angora population was established from a small number of individuals. The flock is closed genetically and thus subsequent loss of genetic diversity is expected. The present population showed an upward trend in the inbreeding coefficient as well as AR while effective population size declined over time and generations. Declining effective population size and increasing inbreeding coefficient is expected to have a negative impact on population and genetic variability. These findings threaten the long-term utilization of this important germplasm. Therefore, it is recommended that there is a need to introduce superior germplasm from outside for optimal selection response to maintain genetic diversity and inbreeding at desirable levels.

## Figures and Tables

**Table 1:** Population structure and level of inbreeding in German Angora rabbit

Items	Whole population	Male	Female
Total number of animals	6,145	3,137	3,008
Number of inbred animals	5,329	2,739	2,590
Number of non-inbred animals	816	398	418
Number of animals with both parent unknown	52	11	41
Number of animals with known parents	6,089	3,126	2,963
Number of animals with progeny	1,045	324	721
Number of animals without progeny	5,100	2,813	2,287
Mean inbreeding coefficient (%)of whole population	8.07	8.10	8.04
Mean inbreeding coefficient (%) of inbred animals	9.30	9.33	9.67

**Table 2:** Inbreeding, average relatedness, and total traced generations traced in German Angora rabbits

Parameter	Mean	Minimum	Maximum
Inbreeding coefficient (%)	08.07±0.07	0	36.96
Average relatedness (%)	15.82±0.06	0.02	21.77
Individual increase in inbreeding (%)	01.13±0.01	0.00	08.51
Equivalent inbreeding coefficient (%)	08.95±0.08	0.00	67.37
Mean maximum generations	10.28±0.07	1.00	22.00
Mean equivalent generations	07.91±0.05	1.00	16.07
Mean complete generations	05.51±0.03	1.00	11.00

**Table 3:** Inbreeding, relatedness coefficient and effective population size for complete generations traced in German Angora rabbit

Generation	N	F (%)	ΔF	POR (%)	FP (%)	AR (%)	N_e_
0	56	0.00	0.00	0.00	0.00	1.92	-
1	160	0.00	0.00	0.00	0.00	5.12	-
2	544	1.12	1.12	30.15	3.72	8.19	44.5
3	737	2.63	1.51	71.64	3.67	10.71	32.7
4	706	5.65	3.02	98.44	5.74	13.67	16.1
5	831	7.84	2.19	100.00	7.84	16.36	21.5
6	722	9.39	1.55	100.00	9.39	18.01	29.8
7	1,035	11.00	1.61	100.00	11.00	19.45	28.1
8	779	11.96	0.96	100.00	11.96	19.97	46.4
9	210	12.86	0.90	100.00	12.86	20.51	48.8
10	302	16.50	3.64	100.00	16.50	21.11	11.9
11	63	21.70	5.20	100.00	21.70	21.15	8.0

F, average inbreeding coefficient; ΔF, rate of inbreeding; POR, percentage of inbred individuals; FP, mean inbreeding coefficient for inbred individuals; N_e_, effective population size; AR, average relatedness.

**Table 4:** Estimates of the increase in inbreeding and effective population size in the German Angora rabbit

Parameter	Method of estimation	Values
Inbreeding increase (%)	Maximum generations	0.85
	Equivalent generations	1.19
	Complete generations	1.85
Effective population size	Maximum generations	58.50
	Equivalent generations	27.05
	Complete generations	42.08
	Individual increase in inbreeding	42.83
	Regression on equivalent generations	38.85
	Log regression on equivalent generations	38.43
	Regression on birth date	39.29
	Log regression on birth date	38.49

**Table 5:** Estimated generation intervals (in years) from various parent-offspring pathways in German Angora rabbit

Pathway	Number	GI (yr)	Standard deviation	Standard error
Sires-buck	313	1.752	1.048	0.059
Sires-doe	677	1.650	0.849	0.032
Dams-buck	313	1.374	0.695	0.039
Dams-doe	679	1.329	0.664	0.025
Overall	1,982	1.513	0.822	0.018

GI, generation interval.

**Table 6:** Parameters characterizing the probability of gene origin in the German Angora rabbit

Parameters	Value
Total number of animals	6,145
Animals with known pedigree (%)	99.09
Number of founders	56
Number of founders actually contributing	52
Effective number of founders (*f_e_*)	18
Number of ancestors contributing	61
Effective number of ancestors (*f_a_*)	16
Number of ancestors explaining 50% of the gene pool (*f_a_*50)	6
Number of ancestors explaining 75% of the gene pool (*f_a_*75)	13
Number of ancestors explaining 100% of the gene pool (*f_a_*100)	61
*f_e_*/*f_a_* ratio	1.12
*f_g_*/*f_a_* ratio	0.39
Effective no. of founder genomes (*f_g_*)	6.22
Effective no. of non-founder genomes (*f_ng_*)	9.50
Genetic conservation index for the whole population	13.91±0.06
Genetic conservation index for males	14.02±0.08
Genetic conservation index for females	13.79±0.09

**Table 7:** Distribution of genetic conservation index (GCI) in the population of German Angora rabbit

GCI	N	N %
1.0 or less	52	0.85
1.1 to 3.0	114	1.86
3.1 to 6.0	514	8.36
6.1 to 9.0	517	8.41
9.1 to 12.0	475	7.73
12.1 to 15.0	680	11.07
15.1 to 18.0	3,733	60.75
18.1 and more	60	0.98
